# Aflatoxin Contamination, Its Impact and Management Strategies: An Updated Review

**DOI:** 10.3390/toxins14050307

**Published:** 2022-04-27

**Authors:** Saba Shabeer, Shahzad Asad, Atif Jamal, Akhtar Ali

**Affiliations:** 1 Crop Diseases Research Institute, National Agricultural Research Centre, Park Road, Islamabad 45500, Pakistan; sabashabbir788@gmail.com (S.S.); asadtaimoor@yahoo.com (S.A.); 2 Department of Biological Science, The University of Tulsa, Tulsa, OK 74104, USA

**Keywords:** aflatoxins, *Aspergillus*, detection, control measures

## Abstract

Aflatoxin, a type of mycotoxin, is mostly produced by *Aspergillus flavus* and *Aspergillus parasiticus.* It is responsible for the loss of billions of dollars to the world economy, by contaminating different crops such as cotton, groundnut, maize, and chilies, and causing immense effects on the health of humans and animals. More than eighteen different types of aflatoxins have been reported to date, and among them, aflatoxins B1, B2, G1, and G2 are the most prevalent and lethal. Early detection of fungal infection plays a key role in the control of aflatoxin contamination. Therefore, different methods, including culture, chromatographic techniques, and molecular assays, are used to determine aflatoxin contamination in crops and food products. Many countries have set a maximum limit of aflatoxin contamination (2–20 ppb) in their food and agriculture commodities for human or animal consumption, and the use of different methods to combat this menace is essential. Fungal infection mostly takes place during the pre- and post-harvest stage of crops, and most of the methods to control aflatoxin are employed for the latter phase. Studies have shown that if correct measures are adopted during the crop development phase, aflatoxin contamination can be reduced by a significant level. Currently, the use of bio-pesticides is the intervention employed in many countries, whereby atoxigenic strains competitively reduce the burden of toxigenic strains in the field, thereby helping to mitigate this problem. This updated review on aflatoxins sheds light on the sources of contamination, and the on occurrence, impact, detection techniques, and management strategies, with a special emphasis on bio-pesticides to control aflatoxins.

## 1. Introduction

Mycotoxins are secondary metabolites produced by different fungal species, mostly *Aspergillus*, *Alternaria*, *Fusarium*, and *Penicillium*. The Food and Agriculture Organization (FAO) has reported 25% contamination of food by mycotoxins worldwide [1]. However, a recent study has shown that 60–80% of crops are infected by mycotoxins worldwide [2], which surpasses the figure given by the FAO. The genus *Aspergillus* contains four subgenera and 339 species [3]. The mycotoxins produced by *Aspergillus* spp. are known as aflatoxins. Aflatoxins are commonly produced by *Aspergillus flavus* and *A. parasiticus*, but some other species, such as *A. nomius*, *A. pseudotamarii*, *A. parvisclerotigenus*, and *A. bombycis* of section *Flavi*; *A. ochraceoroseus* and *A. rambellii* from section *Ochraceorosei;* and *Emericella astellata* and *E. venezuelensis* from *Nidulatans*, have also been reported as aflatoxin producers [4]. Several types of aflatoxin have been reported, and their contamination of economically important crops and food is a major concern worldwide [5]. They are carcinogenic as well as mutagenic in nature and cause aflatoxicosis in both humans and animals [6]. Recently, a coronavirus disease associated with pulmonary aspergillosis has also been reported. It is recognized that pulmonary aspergillosis increases the severity of corona in immunocompromised patients. In total, 20 cases of Coronavirus disease-associated pulmonary aspergillosis (CAPA) have been reported worldwide [7]. Due to their ubiquitous nature, about 4.5 billion of the world’s population is subjected to aflatoxin contamination. Owing to the adverse effect of aflatoxins on living organisms, their maximum limit in food and feed products for the consumption of humans and animals is set to 20 ppb (parts per billion) by the European Commission and the U.S. Food and Drug Administration [8,9], and 4 ppb by European Union [10]. 

Currently, more than 18 different types of aflatoxins (Table 1) have been discovered, but the most common and important ones are aflatoxins B1, B2, G1, and G2 [11,12]. Their prevalence in food makes them more important than the other types. Additionally, aflatoxin B1 binds with DNA and alters its structure [13], causing genotoxicity. Aflatoxins B1, B2, G1, and G2 were given these names due to their characteristic of absorbing and emitting light. Therefore, B1 and B2 show blue fluorescence under ultraviolet light at 425 nm, while G1 and G2 appear green under U.V. light at 540 nm [6]. *Aspergillus bombycis*, *A. nomius*, *A. parasiticus*, *A. parvisclerotigenus*, *A. pseudocaelatus*, *A. minisclerotigenes*, and *A. arachidicola* produce all four types of aflatoxins, viz. B1, B2, G1, and G2; whereas, *A. flavus*, *A. ochraceoroseus*, and *A. rambellii* only produce Aflatoxin B1 and B2; and *Aspergillus pseudonomius*, *A. pseudotamarii*, *Emericella astellata*, *E. olivicola*, and *E. venezuelensis* only produce aflatoxin B1 [14]. Aflatoxin B1 is most commonly related to aflatoxicosis, as well as acute toxicity, chronic toxicity, carcinogenicity, teratogenicity, genotoxicity, and immunotoxicity [15]. Aflatoxin M1 and M2, which are derivatives of aflatoxin B1 and B2, respectively, have been found in animal urine and milk [16]. Aflatoxins B1 and M1 were declared human Group 1 and Group 2B carcinogens, respectively, by the International Agency for Research on Cancer (IARC) in 1987 [17,18]. 

*A. flavus* is a very common soil fungus, found worldwide, but its existence is more common in subtropical and tropical climates. *A. flavus* is mostly found in latitudes between 16° and 35° and is rare above 45° latitude. *A. flavus* survives as sclerotia or conidia in soil for up to three years and as mycelia in infected plant tissues. These sclerotia overwinter in the soil during harsh environmental conditions. Upon favorable environmental conditions, sclerotia germinate into mycelia and then produce conidiophores that are dispersed through the air and infect a range of crops. 

Temperature, humidity, environmental stress, injury caused by insects or birds on the host, and post-harvest practices are some of the factors that are involved in the growth, colonization of a host by *A*. *flavus*, and toxin production [19]. In field conditions, high temperature with a period of drought favors the production of aflatoxins [20]. The infection of different crops with *A. flavus* may cause symptoms such as ear or boll rot, and yellow mold may also occur asymptomatically. Common hosts infected by *A. flavus* at pre-harvest stages include maize, groundnuts, chili, cottonseed, and tree nuts, while wheat, sorghum, and rice are more susceptible at post-harvest stages. Improper handling and storage conditions of crops greatly influence the contamination of crops by *Aspergillus* spp. at the post-harvest stage [21].

## 2. Aflatoxin Contamination

Abiotic factors such as temperature, water activity, pH, carbon, and nitrogen have a great influence on the aflatoxin biosynthesis pathway [22], but in particular, aflatoxin contamination is highly dependent upon temperature and water activity. These two conditions not only encourage the growth of aflatoxin producing fungi, mainly *A. flavus*, but also have a great effect on the activation of the aflatoxin-producing gene cluster [23,24,25]. Higher water activity favors better fungal growth and toxin synthesis [26]. It is estimated that a water activity of approximately 0.99 a_w_ and a temperature of 29–30 °C encourage aflatoxin production [25]. Both of these factors, viz, temperature and water activity (a_w_), play a key role in the transcription of two important regulatory genes (aflR and aflS) in the aflatoxin biosynthesis pathway [22]. Temperatures below 25 °C and above 37 °C are not conducive for the growth and production of aflatoxins, whereas moisture levels below 0.85 a_w_ slow down the growth and production of toxins, and it completely stops at 0.70–0.75 a_w_ [26].

As stated by the RASFF (Rapid Alert System for Food and Feed) database, in the year 2020 [27], most of the aflatoxin contamination was reported in peanuts, rice, nuts (pistachios, hazelnuts, and almonds), spices, and dried figs, with up to 1000 μg/kg. This high concentration was mainly due to the poor food management practices during the current situation of the COVID-19 pandemic. This will ultimately result in a greater intake of aflatoxin-contaminated food by animals and humans. Therefore, an increase in health concerns related to it can also be expected [28]. Aflatoxin contamination is most prevalent in Asia and Africa, where climatic conditions favor the development of aflatoxigenic strains in both field and storage conditions. China is also facing the problem of aflatoxin contamination in agricultural commodities. Owing to global climate change, aflatoxin is an emerging threat in regions that were previously free from this menace. Recently, there have been a few reports of aflatoxin in different regions of Europe [29]. 

## 3. Impact on Human and Animal Health

Aflatoxins are considered not only hazardous for humans but also animals. They can cause different acute and chronic illnesses, which are discussed below. 

### 3.1. Aspergillosis 

Aspergillosis is a lung infection caused by *Aspergillus* species in immunocompromised individuals. It is caused by twenty different species of *Aspergillus*, but *A. fumigatus* and *A. flavus* are the main agents of aspergillosis in both humans and animals. Worldwide, most cases of aspergillosis infection in humans are caused due to excessive inhalation of *Aspergillus* spores, while the second main cause of infection is the transmission of spores through infected wounds, as well as through the smoking of contaminated tobacco or marijuana plants. Different animals such as rabbits, chickens, turkeys, and geese are also infected by aspergillosis. In addition, *A. flavus* also causes stone brood disease in honeybees. Clinically, aspergillosis has different forms, which include extrinsic asthma, allergic bronchopulmonary aspergillosis, extrinsic allergic alveolitis, saprophytic pulmonary, and extra-pulmonary colonizing, as well invasive pulmonary and extrapulmonary aspergillosis [19]. Allergic bronchopulmonary aspergillosis (ABPA) accumulates in 1–15% of the world’s population already infected with cystic fibrosis and also in 2.5% of asthma patients, which in total comprises 4.8 million people globally. Out of the 4.8 million of the world’s population affected with ABPA, 400,000 people are also affected with chronic pulmonary aspergillosis (CPA). On the other hand, 1.2 million people with tuberculosis are also co-infected with chronic pulmonary aspergillosis (CPA) [30,31,32]. Aspergillosis ranks in the list of the top four diseases that cause death in immunocompromised patients worldwide [33]. Although *A. flavus* does not cause aspergillosis often, the rare cases of infection can be very severe. In North America, around 65% of aspergillosis in children is caused by *A. flavus*. Moreover, it is also the main causative agent of mycotic keratitis [19]. 

### 3.2. Aflatoxicosis

Aflatoxicosis is the poisoning associated with the extensive consumption of *Aspergillus* species, mainly *A. flavus* in the form of spores or contaminated food that can cause chronic or acute aflatoxicosis in humans and animals. Chronic aflatoxicosis includes liver cancer, human hepatic cell carcinoma, stunted growth, reduced immunity, and cirrhosis in malnourished children; acute aflatoxicosis includes high fever, vomiting, ascites, liver failure, edema of feet, and jaundice with a high mortality rate compared to chronic aflatoxicosis [34]. Accurate values of the aflatoxin concentration that causes aflatoxicosis have not been confirmed; however, with the help of a few studies, it is estimated that generally 1000 μg/kg of aflatoxin concentration in food can cause aflatoxin toxicity in humans [35]. In the case of animals, a tolerable amount is 50–300 μg/kg [36]. Major outbreaks of aflatoxicosis were reported in India and Kenya in 1974 and 1981, respectively. It is worth mentioning that 500 cases and 200 deaths have occurred due to aflatoxicosis worldwide since 2004 [6]. 

### 3.3. Cancer

Aflatoxins are reported as a Group 1 carcinogen and their long-term exposure may cause kidney, liver, lung, or colon cancer in both animals and humans. In Africa and Asia, the primary liver cancer known as hepatocellular carcinoma is related to aflatoxin B1, while about 4.6–28.2% of hepatocellular carcinoma around the world is reported to be caused by aflatoxin consumption [37]. Moreover, aflatoxin B1, which is characterized as a Group 1 carcinogen, is found to be hazardous if a concentration of 20–120 μg/kg is consumed per day for 1 to 3 weeks [35]. However, the extent of aflatoxin toxicity highly depends upon the immunity of the host [13]. Hepatocellular carcinoma (HCC) is the major outcome of aflatoxin exposure and is the cause of 75–85% cases of liver cancers worldwide. Furthermore, 1480 new cases of liver cancer due to aflatoxins were identified in Tanzania in 2016 [38]. 

## 4. Detection of Aflatoxin-Producing Strains

A variety of different detection methods for aflatoxin contamination has been used, and includes cultural and molecular-based techniques, which are discussed below. 

### 4.1. Culture-Based Techniques 

Different culture media can be used to morphologically differentiate between toxigenic and atoxigenic strains of *A. flavus*. These media include coconut agar medium (CAM), coconut milk agar (CMA), yeast extract sucrose (YES) medium, and aflatoxin producing ability (APA) media. The culture of *A. flavus* is grown on CAM, CMA, YES, or YES mediated with methyl β-cyclodextrin, and APA, as described previously by [21,39,40,41,42]. When the fully grown cultures of *A. flavus* on CAM, CMA, and YES media amended with 3% methyl β-cyclodextrin are observed under UV light of 365nm wavelength, the toxigenic isolates show a fluorescent ring that surrounds the colony, while the atoxigenic isolates show no fluorescence. 

On APA medium, the blue fluorescence is observed in toxigenic isolates; while no fluorescence is seen in atoxigenic isolates when visualized under UV light (365 nm wavelength). When cultures on YES medium are subjected to ammonia vapors, the ammonia fumes cause the toxigenic isolates to change color from pink to red or plum, while atoxigenic isolates do not change their color. This change in color is due to the presence of seven yellow pigments in toxigenic isolates of *A. flavus*, named as norsolorinic acid, averantin, averufin, versicolorin C, versicolorin A, versicolorin A hemiacetal, and nidurufin, which play a role as aflatoxin biosynthesis mediators. These pigments act as pH indicator dyes and, hence, upon reaction to ammonia the pH increases, which results in a change of color. Only norsolorinic acid pigment changes its color from red to plum in high pH, while the other six change their colors from pink to red as investigated and described by [43]. 

**Figure 1:**
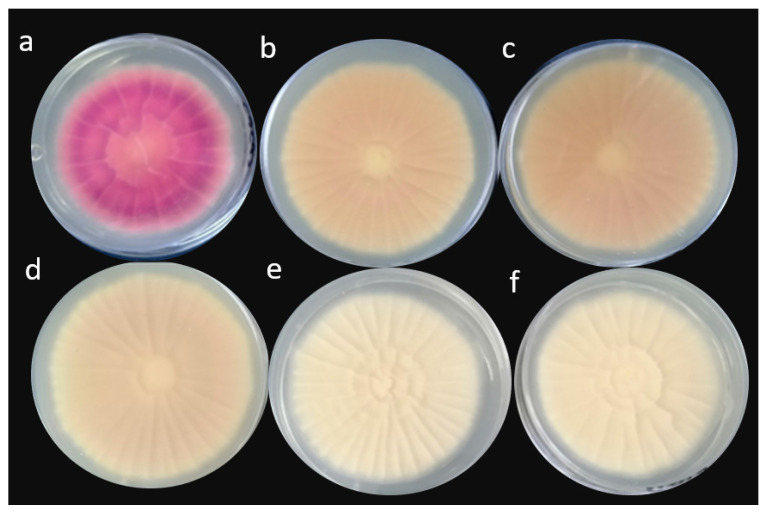
Cultures of *Aspergillus flavus* after exposure to ammonia vapors. (**a**) Cultures showing a highly toxigenic strain, based on their change in color to dark plum after exposure, (**b**–**d**) cultures showing moderately toxigenic stains based on their change in color to light reddish to pinkish color after exposure, (**e**,**f**) cultures showing atoxigenic strains with no change in color (Saba and Atif, unpublished data).

The difference between toxigenic and atoxigenic strains can be seen in Figure 1, after exposure to ammonium hydroxide. Culture-based detection techniques are only used to differentiate between toxigenic and atoxigenic fungi, owing to the reason stated above; therefore, aflatoxin concentrations cannot be quantified through this procedure. For quantitative studies of aflatoxin, other methods can be used, which include ELISA and chromatographic techniques. 

### 4.2. Molecular Based Techniques 

Molecular-based techniques are more reliable than culture-based techniques. They involve the use of different markers to amplify the genes that take part in the biosynthesis pathway of aflatoxins. Multiplex and real-time PCR assays have been developed to amplify the different genes that are involved in aflatoxin biosynthesis pathways, such as *nor*-1, *apa*-2, *omtA*, *ver*-1, *aflRS*, *aflJ*, and *omtB* genes. These genes are amplified by using three different systems. In the first system, genes *nor*-1, *omtA* (*omt*-1), and *apa*-2 are amplified; in the second system *nor*-1, *omtA* (*omt*-1), and *ver*-1 are targeted; while in the third system, PCR amplifies the *omtB*, *aflRS*, and *aflJ* genes [44]. Table 2 shows the PCR primers used to amplify different aflatoxin biosynthesis genes.

## 5. Quantification/Detection of Aflatoxin

### 5.1. Immunochemical Methods for Detection of Aflatoxin

These methods include enzyme-linked immunosorbent assay (ELISA), radioimmunoassay (RIA), and immunodipsticks (lateral flow devices) that use antigens and antibodies and depend on their binding specificity. ELISA is considered a rapid and suitable method for the detection of aflatoxins in crops and food products. It is commonly used in research and medical laboratories, and many ELISA kits are commercially available. Specific three-dimensional structured aflatoxin is differentiated by a specific antibody [62]. Moreover, the antigens or antibodies can be labeled by enzymes that can be analyzed using specific substrates, to increase the sensitivity of ELISA. This technique is not only cheaper than the others, but also easy to use [63,64]. ELISA kits such as Veratox^®^ are widely used for the quantification of aflatoxin in different samples and can detect aflatoxin concentrations in the range of 5–50 ppb [65]. 

In contrast, radioimmunoassay (RIA) was the first method that was developed and used to detect insulin in human blood. It has also been used for the detection of aflatoxins in food. The principle of this technique depends on the binding of a labeled antigen with an unlabeled antigen. These two bonded antigens then react with the limited quantity of antibody; therefore, this method is also referred to as ‘limited reagent assay’. A radioimmunoassay is highly specific and sensitive and requires a smaller amount of sample, but is considered dangerous because of the use of a radioactive-labeled antigen; therefore, this technique is not very commonly used today [66]. 

Apart from these, immunodipsticks (lateral flow devices) are also used. Immunodipsticks are lateral flow devices that use immunochromatographic techniques to carry highly sensitive and specific reactions between antibody and antigen for the detection of aflatoxin B, G, and M1. In lateral flow immunoassay (LFIAs), lateral flow devices are used that contain a porous membrane that is composed of nitrocellulose, an absorbent pad composed of cellulose, a sample pad of glass fiber, and a rigid backing. Lateral flow devices use antigens that are gold coated, which gives red-colored binding zones. The sample is added as a liquid in the sample pad section of the device, in which the dipsticks are directly immersed. The sample from the sample pad flows through the membrane towards the absorbent pad, where the aflatoxins bind with the gold particles that were suspended by the sample, giving it a red color. The technique is rapid and easy to use; however, it is not cost-effective [67].

### 5.2. Biosensor-Based Techniques 

Biosensors use antibodies or antigens to recognize different biological components. Their binding to complementary species is detected through the graphite, carbon, or gold that is attached as a signal transducer. Piezoelectric quartz crystal microbalances (QCMs) are highly sensitive unlabeled devices that can directly detect antigens. When an antigen comes in contact with an antibody, it is confined on the surface of the quartz crystal, which alters the mass of the electrode surface, upon which the phenomena of piezoelectric quartz crystals depend. The concentration of antigen and antibody complex that is confined on the surface of a quartz crystal is directly proportional to the change in mass of the electrode surface; hence, this principle allows detecting and quantifying the immune complex. QCMs have been successfully used to detect aflatoxin B1 [68].

### 5.3. Optical Immunosensor 

Different optical immune sensors are used for the detection of aflatoxins. The most common ones are surface plasmon resonance detection (SPR) and optical waveguide light-mode spectroscopy (OWLS). SPR is a well-known principle that allows the real-time detection of the antibody–antigen interface. This technique has been commonly used to detect toxins, nucleic acids, cells, peptides, proteins, biomarkers, genes, etc. SPR devices are large and heavy. Therefore, their use in field conditions is not possible. Recently, a palm sized SPR device has been developed [69] for the on-site detection of aflatoxin B1 in infected grains. Many other researchers have also used these kinds of mini SPR devices for the detection of different chemical and biological species [70]. Moreover, a novel SPR sensor has also been established [71] that uses nanoparticles incorporated on a gold chip to detect aflatoxin B1. In OWLS, the polarized laser light angle that is diffused through grating is accurately measured and incorporated into a narrow waveguide. Photodiodes detect the intensity of this light incorporated into the waveguide. Different mycotoxins have been detected, including aflatoxin B1 in the range of 0.5–10 ng/mL in wheat and barley samples, as well as ochratoxins A through OWLS [70].

### 5.4. Electrochemical Immunosensors 

Electrochemical immunosensors are considered simple, cheap, and timesaving for the detection of aflatoxins. The amplifiers in electrochemical immunosensor devices recognize the signals produced by the integration of antibodies into a biorecognition layer, which can be quantified. These signals are produced as a membrane potential in response to the binding of ions to a sensor membrane. The difference in membrane potential is then evaluated. Different electrochemical immunosensor devices have been used to detect aflatoxins that rely on the restriction of antibodies on the surface of an electrode, but many devices also use enzymes as biological agents to produce signals for the detection of aflatoxins. Moreover, a few non-enzymatic electrochemical immunosensors have also been developed for aflatoxin analysis [70,72]. 

Recently another electrochemical immunosensor technique has been established to detect aflatoxin M1 in milk. This technique can detect concentrations of AFM1 from 0.01 to 1 μg/L. It uses biosensors that are constructed by printing electrodes that work along with the single-walled carbon nanotubes (SWCNTs) that are layered with particular antibodies. This method is cost effective, quick, and easy to use, and additionally, the printing material is flexible; hence, any printing material can be used. For instance, insulators, conductors, and semiconductors [73].

### 5.5. Sensor Array Method

Sensor array methods, including head space sensor arrays, are a rapid method to detect toxins in a sample. This method of detection uses a range of electrochemical sensors. A variety of semiconducting devices are used, on which a broad spectrum of reactions occur that produce signals. The array of electrochemical sensors converts those signals into data that can be evaluated using different statistical software. This method has been successfully used to detect aflatoxin M1 in milk samples [74].

### 5.6. Microfluidic Sensor Method 

The microfluid sensor method is used to detect aflatoxin M1 and B1. In this technique, a complex of aptamer, along with a probe that has gold nanoparticles (AuNPs), is surface assimilated on a paper-based microfluidic device (μPAD). The sample is allowed to run on this paper-based device (μPAD). After that, a quick change in color can be observed, both with naked eye, and by using spectroscopy and capillary techniques. By using this technique, the aflatoxins M1 and B1 can be detected between the range of 1 µM to 1 pM, with a limit of up to 10 nM. This method of detection is precise, quick, and economical and can be used for on-site detection of aflatoxin in milk samples [75,76]. Another aptamer sensor-based technique has been recently used to detect aflatoxin M1 in the range 0.0005–0.8 μg/L, using a complex of graphene oxide with gold nano-particles (AuNPs) fabricated with a pencil graphite electrode (PGE) [77].

### 5.7. Chromatographic Methods

Chromatographic methods use two phases, viz. a mobile phase (mostly liquid) and a stationary phase (liquid or solid), and depend upon the physical interaction between these two phases. Three chromatographic techniques that are widely used are thin-layer chromatography (TLC), high-performance liquid chromatography (HPLC), and gas chromatography (GC). TLC is the most commonly used confirmatory method for the detection of aflatoxins in agricultural commodities, plants, and food products, while HPLC is a commonly used method for the determination of organic compounds. Gas chromatography (GC) is not very common, because it’s expensive and also requires the proper cleaning of equipment before every analysis. TLC contains a stationary phase that is silica, cellulose, or alumina, which is immobilized on a matrix that is either made of glass or plastic, while the mobile phase consists of a mixture of methanol, acetonitrile, and water that moves on the stationary phase and carries the sample along with it. TLC is useful to detect different types of mycotoxins in one test, and it is highly sensitive and requires expert technicians. Moreover, its precision is not up to the mark, due to which another alternative technique, known as high-performance thin-layer chromatography (HPTLC), has been developed, which is an efficient and highly precise method [78,79]. HPLC consists of a stationary phase that is attached to a glass or plastic tube, and a mobile phase that consists of either aqueous or organic solvents. The sample runs along both mobile and stationary phases and distributes between both phases, depending upon the affinity of the sample for both phases and the rate of its flow. Different detectors, such as a fluorescent detector (FLD), ultraviolet (UV) detector, or diode array detector (DAD) are used in HPLC to detect aflatoxins. HPLC gives rapid and precise results, but HPLC equipment is very expensive and can only be operated by an expert person [80]. 

The gas chromatography (GC) technique uses gas as the mobile phase, while a liquid is confined to solid particles as a stationary phase that is restricted in a column of glass tube or stainless steel held at a suitable temperature. The sample is converted into a gas through vaporization and carried along the gas phase through the stationary phase. The different components of samples will then be separated between the stationary and mobile phases. After the distribution of components, the volatile agents are detected with an electron capture detector (ECD) or a flame ionization detector (FID) and mass spectrometer (MS) [81]. 

Many studies have shown the successful use of different chromatographic methods, to detect aflatoxin concentration in milk, dairy products, wheat, maize, peanuts, cereals, dried figs, coffee beans, spices, and adult and baby food [82,83,84,85,86,87,88]. TLC is considered the most commonly used method, but high-performance liquid chromatography (HPLC) and liquid chromatography-mass spectrometry (LC–MS) have been reported as the best techniques for the quantification of aflatoxins in food commodities, due to their high sensitivity and accuracy [89]. A study demonstrated the use of a high-performance liquid chromatography-mass spectrometry (LC–MS) technique to analyze the aflatoxin concentration in baby food in the range 0.003–0.008 μg/L [90]. In milk samples, high performance liquid chromatography (HPLC) coupled with mass spectroscopy or fluorescence detection is reported as a reliable technique for estimating aflatoxin concentrations [91].

### 5.8. Spectroscopic Methods

Spectroscopic methods include fluorescence spectrophotometry, which detects aflatoxins using fluorometry in which different molecules fluoresce by emitting energy at specific wavelengths. Through fluorescence, spectrophotometry aflatoxins can be quantified in the range of 5 ppb to 5000 ppb in less than five minutes [92]. The other method of spectroscopy is frontier infrared spectroscopy, which uses infrared radiations to irradiate the molecules and then measure the vibration of bonds in molecules. As the size, length, and strength of bonds vary in molecules, the absorbance of radiation also varies from bond to bond, and, hence, their frequency of vibration also differs [93].

## 6. Control of Aflatoxin Contamination

Due to the adverse effect of aflatoxins on humans and animals worldwide, the control of aflatoxins is considered mandatory. For this purpose, different control strategies, including biological, chemical, and physical management practices, are being used worldwide and are discussed below. 

### 6.1. Biological Control

Biological control involves the use of bio-pesticides made of atoxigenic strains that lack the ability for producing aflatoxin, thereby reducing aflatoxin in field conditions. Use of different bacterial or fungal isolates can also help in controlling aflatoxin. 

#### 6.1.1. Atoxigenic *Aspergillus* Strains against Toxigenic *Aspergillus* Strains

The most potent method used for controlling aflatoxin in the field is the use of naturally occurring atoxigenic strains through competitive exclusion. Based on morphology, strains of *A. flavus* are divided into S and L strains. S strains produce small sclerotia but a high level of aflatoxins, whereas L strains produce large sclerotia but a lower level of aflatoxin, and even consist of atoxigenic strains that cannot completely produce aflatoxins, due to some genetic variations [94]. The use of atoxigenic strains of *Aspergillus* against toxigenic strains has successfully controlled the aflatoxin production in the field, which ultimately controls the aflatoxin contamination in storage conditions. This strategy of using atoxigenic *Aspergillus* strains against toxigenic strains was first performed by Cotty and Bayman [95], and, subsequently, it has been employed for the control of aflatoxin around the globe (Table 3). Atoxigenic strains have been used against toxigenic strains as biocontrol agents for almost two decades [96]. 

**Figure 2:**
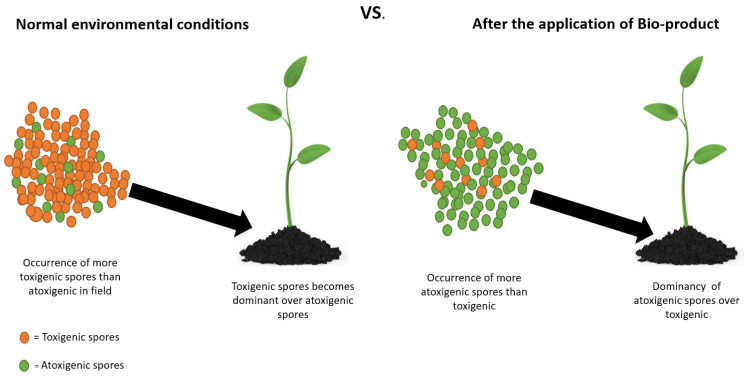
Schematic diagram showing the principle of biocontrol pesticides. During normal environmental conditions, usually ratios of toxigenic spores are higher than the atoxigenic spores present in the field. After the application of bio-pesticide, the atoxigenic spores are increased and competitively exclude naturally occurring toxigenic strains.

Successful biological control through atoxigenic strains requires the presence of a high ratio of atoxigenic strains in the field, as compared to toxigenic strains (Figure 2) [96]. In addition, it is to be noted that the application of atoxigenic biopesticides has an inverse relation with aflatoxin accumulation, and it does not escalate the quantity of *A. flavus* [109]. Biopesticide (atoxigenic strain) does not produce aflatoxins, because of a deletion in the gene or of genes that are involved in the aflatoxin biosynthesis pathway. However, in other exceptional cases, such as in the case of AF36, a SNP (single nucleotide polymorphism) initiates a stop codon in the aflC (pksA) gene that plays a role in the polyketide pathway of aflatoxin synthesis and, hence, causes the AF36 strain to stop producing aflatoxins [110]. Furthermore, when the biopesticide is applied in the field, it competes with toxigenic strains; hence, restraining the multiplication of toxigenic fungi and ultimately reducing aflatoxins [111]. 

The use of atoxigenic strains as biocontrol agents requires prompt characterization of atoxigenic isolates, to assess their stability, adaptation, and efficacy under different environmental conditions. For this purpose, the large deletions in the aflatoxin gene clusters present in potential atoxigenic strains are evaluated. This aflatoxin gene cluster is present on the chromosome III of the genome, comprising of 32 different genes. These genes take part in the synthesis of aflatoxins [112]. In the beginning, to detect the deletions in aflatoxin clusters, 32 different PCR markers for 32 separate amplifications were required, which were reduced to only four with time [113]; however, this resulted in a lack of information, with no thorough knowledge about the cyclopiazonic acid (CPA) gene and the sub-telomeric region. 

Later, Callicott and Cotty [114] developed four multiplex PCR amplifications to amplify 32 markers with detailed information about all four regions, viz. sugar cluster, aflatoxin cluster, cyclopiazonic acid (CPA) cluster, and sub-telomere region, which not only gave a detailed insight into the characterization of fungal structure but also helped in the monitoring of the atoxigenic potential of *Aspergillus* spp. Currently, this technique of multiplex PCR, known as Cluster Amplification Pattern (CAP) analysis, is widely used for the rapid characterization of atoxigenic strains, to verify their biocontrol potential. In CAP analysis, 32 markers, along with an internal amplification control (iac), are placed at regular intervals, to monitor the deletion in the genes for the differentiation of atoxigenic isolates from toxigenic (Table 4).

In Arizona, USA, the strain AF36 was registered as the first biocontrol agent of aflatoxin contamination in cottonseed. This biocontrol agent also showed effective results against toxigenic strains of *A. flavus* producing aflatoxins in corn. About a 70% to 90% reduction in aflatoxin production in peanut and cotton has been achieved in field experiments by using atoxigenic strains of *Aspergillus* [97]. 

Other strains, such as strain NRRL21882 of *A. flavus* and strain NRRL21369 of *A. parasiticus*, that are applied in field conditions are found to be very effective against aflatoxin contamination, both in pre-harvest and as post-harvest stages in peanuts. The strain NRRL21882 of *A. flavus* is also available commercially as a biopesticide, named Afla-guard [98]. Atoxigenic strain BN30 is successfully used in the control of aflatoxin contamination of maize in Africa [106]; similarly, in Australia, the use of atoxigenic strains was found to reduce aflatoxin contamination in peanuts by 95%, as reported by [115]. Additionally, in China, 30 atoxigenic *A. flavus* strains have been tested, out of which strain AF051 showed high effectiveness in the control of aflatoxin production, by up to 99%, in peanut fields [106]. 

**Figure 3:**
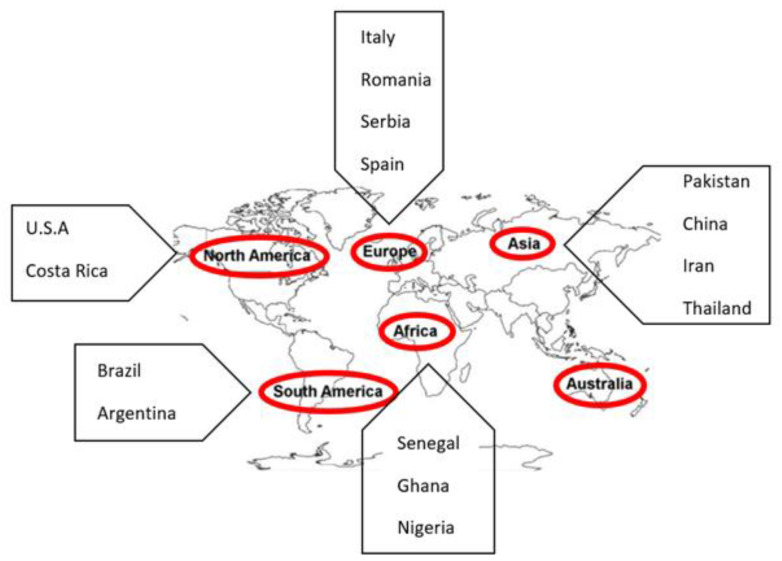
Map showing the countries where bio-pesticide is under the process of being developed. It includes Pakistan, China, Iran, and Thailand in Asia; Senegal, Ghana and Nigeria in Africa; Italy, Romania, Serbia, and Spain in Europe; U.S.A and Cost Rica in North America; Brazil and Argentina in South America; and in Australia.

A four-year study conducted by [99] showed a decrease of aflatoxin contamination in maize of up to 65–94% using atoxigenic CT3 and K49 strains in the southern U.S. Likewise, another two-year study demonstrated the successful use of atoxigenic AR27, AR100G, and AFCHG2 strains of *A. flavus* against toxigenic strains in the groundnut fields of northern Argentina [104]. Apart from *A. flavus* and *A. parasiticus*, the FS10 strain of *Aspergillus niger* has also shown a high rate of reduction in the production of aflatoxins in the field [105,116]. Biopesticides for controlling aflatoxin are being used in different parts of the world. Moreover, some countries are on the brink of registering biopesticides in their respective countries. Figure 3 shows the regions where the use of atoxigenic strains for aflatoxin mitigation is completed or under process. 

#### 6.1.2. Biological control at the experimental stages

The following micro-organisms are being used in experimental trials against fungi producing aflatoxins. Most of the studies mentioned in this section that were conducted to evaluate the ability of pathogens as biocontrol agents were limited to study trials, and have not yet been prepared as a bioproduct; therefore, there are no bacterial or fungal strains that are commercially available as a biopesticide specifically for the control of aflatoxins [94]. However, some studies have shown that most of the bio-fungicides that are not specific to aflatoxins also show a reduction in the conidial production of *A. flavus* [117]. 

##### Biological Control with Yeasts

Several yeast strains, including *Debaryomyces hansenii* strain BCS003 (marine yeast), *D. hansenii* (native yeast), *Kluyveromyces* spp., *Pichia anomala*, *Candida maltose*, *Saccharomyces cerevisiae* RC008, and *Saccharomyces cerevisiae* RC016 show a drastic effect on the production of aflatoxins, as well as the growth of *Aspergillus* spp. [94]. These species still need to be tested in field conditions and made applicable in vivo.

##### *Trichoderma* spp.

*Trichoderma* spp. is considered an effective biocontrol agent against many fungal species. The species of *Trichoderma* that are found to be highly effective against aflatoxins are *T. harzianum* and *T. viridae*, with the inhibition rate of aflatoxins being greater than 80% [118]. Two other species, *T. longibrachiatum* and *T. auroviride*, also reduced the aflatoxin levels in the field, as well as in the greenhouse, by 50% [94]. It was also reported that *Trichoderma* spp. successfully reduced the aflatoxin contamination in groundnut and sweet corn, by up to 57% and 65%, respectively [94]. 

##### *Penicillium* spp.

*Penicillium* specie *P. chrysogenum* strain RP42C produces a protein that suppresses the growth of toxigenic *Aspergillus* strains [119]. Likewise, *P. nalgiovense* is considered a common biocontrol agent against many plants and pathogenic fungi, as well as the secondary metabolites produced by them [94].

##### Biological Control with Bacteria

Many bacterial species have shown successful inhibition of aflatoxin production in-vitro by inhibiting the growth of *Aspergillus* species. These bacterial species include different species of *Lactobacilli*, *Pseudomonas*, *Ralstonia*, *Burkholderia*, *Streptomyces*, *Stenotrophomonas*, and *Bacillus*.

(a) *Bacillus* spp.

Among all of the beneficial bacteria, *Bacillus* spp. is the most studied pathogen for the control of aflatoxins. During a study conducted by Kong [120], *Bacillus megaterium* prevented the production of aflatoxins in broth medium by 100%. Another study revealed that *Bacillus subtilis* can control the growth of *Aspergillus parasiticus* up to 92% and the production of aflatoxins by up to 100% [121]. Among different *Bacillus* species, *B. megaterium*, *B. subtilis*, *B. amyloliquefaciens*, *B. mojavensis*, B. cereus, *B. mycoides*, and *B. pumilus* are considered the most effective against aflatoxin contamination [94].

(b) *Pseudomonas* spp.

*Pseudomonas* is the most prevalent pathogenic group in soil. When *Pseudomonas fluorescens* was tested in peanut medium against aflatoxins, it showed a 99.4% inhibition of aflatoxin B1 produced by *A. flavus* [122], along with a reduction in the germination of *A. flavus* conidia up to 20% [123]. Different strains of *P. chlororaphis* obtained from maize soil reduced the growth of *A. flavus* by 100% [94]. Moreover, another study conducted by [124] demonstrated an inhibition of aflatoxins up to 82.9%, as well as the reduction in the growth of *A. flavus* by up to 68.3% using the *P. protegens* strain AS15 obtained from rice grains. Apart from these, many other strains of *Pseudomonas* have shown successful inhibition of aflatoxin contamination and growth of A. flavus in various media [94].

(c) *Lactobacillus* spp.

*Lactobacillus*, also known as lactic acid bacteria (LAB), is the group of bacteria that produce lactic acid through fermentation. These bacteria are being widely used in food technology. In this group of bacteria, several species, such as *L. delbrueckii subsp. Lactis*, *L. reuteri*, *L. plantarum*, *L. acidophilus*, *L. paraplantarum*, *L. rhamnosus*, *L. fermentum*, *L. pentosus* and *L. casei* were found to be effective against aflatoxins. However, among all lactobacillus species, *L. plantarum* was demonstrated to be the most effective biocontrol agent against aflatoxin-producing fungi [125,126,127].

(d) *Streptomyces* spp.

Some of the *Streptomyces* species, including *S. yanglinensis*, *S. anulatus*, *S. alboflavus* and *S. roseolus*, showed very good results when used against aflatoxigenic fungi as biocontrol agents. A strain of *Streptomyces* was successfully able to completely control the growth and conidial production of *Aspergillus flavus*. Likewise, strain ASBV-1 reduced the production of aflatoxins in groundnut [94].

(e) Other *Bacterial* spp.

The bacterial strain *Serratia marcescens* JPP1, obtained from peanut shells, is an endophytic beneficial bacterium that remains asymptomatic and reduces the production of aflatoxins by up to 98%, as well as inhibiting the growth of *A. parasiticus* by up to 95% [128]. Another bacterial species, *Nannocystis exedens*, can inhibit the growth of both *A. parasiticus* and *A. flavus*. During a study, 171 different bacterial isolates, including the species *Pseudomonas*, *Delftia acidovorans*, *D. acidovorans*, *Achromobacter xylosoxidans*, *Burkholderia cepacia*, *B. pyrrocinia*, *Ralstonia paucula* and *Bacillus*, were found to be effective biocontrol agents against *A. flavus* [94].

### 6.2. Chemical Control 

For chemical control, different organic and inorganic acids, which include citric acid, lactic acid, tartaric acid, propionic acid, and hydrochloric acid, have shown good results for the control of aflatoxins, where citric acid and lactic acid were more effective than the others, with inhibition rates up to 86–92% and 67%, respectively. The chemical sodium bisulfite can control aflatoxins at different rates, depending upon the technique with which it is used. For instance, a 28% aflatoxin control rate can be achieved when used at 25 °C, 65% when 0.2% H_2_O_2_ is applied 10 min before sodium bisulfite, 48% when 45 °C heat was applied for up to 1 h after the application of sodium bisulfite, and a 68% control rate was accomplished when 65 °C heat was applied after application for 1 h. Ammonium persulfate, which is an oxidizing agent, showed a 31–51% reduction in aflatoxin contamination. Sodium hydrosulfite is also considered highly effective, with a reduction rate of 96–100% when applied in the range of 0.25–2%. 

Moreover, treatment with some other salts and acids, as well as alkaline compounds, including chloride acid, phosphoric acid, sodium, potassium, calcium hydroxide, sodium bicarbonate, sodium chloride, and sodium sulfate also causes a reduction in aflatoxin contamination by up to 18–51%. Several studies have also shown the successful use of ozone and chitosan nanoparticles for the reduction of aflatoxin content [129]. Different fruit derivatives such as hexane and chloroform can also be used to inhibit the production of aflatoxins. Antioxidants such as butylated hydroxyanisole, butylated hydroxytoluene, and propylparaben showed remarkable results in the suppression of *A. flavus*, leading to a reduction in aflatoxin contamination [106]. 

Another chemical control method that can be used for the mitigation of aflatoxins is the use of adsorbents. In this method, different adsorbents, such as synthetic polymers, including polyvinyl pyrrolidone and cholestyramine; zeolites; complex carbohydrates, including polysaccharides and cellulose; activated charcoal; alumino, including clay, yeast, bentonite, diatomaceous earth; and active carbon can be used. These adsorbents are included in broiler diets, and during digestion, the toxins attach to these adsorbents and, hence, prevent the mixing of toxins into the blood, and they are later removed from the body [129,130].

### 6.3. Physical Control

Different physical methods can be used to control aflatoxin contamination in different food commodities. These methods may include mechanical sorting, heat inactivation or thermal treatment, irradiation, density segregation, etc. Aflatoxins are resistant against heat treatments, but decontamination through heat is reported to have been successfully used in the case of dry fruits and nuts. For example, in the case of almonds, roasting at 200 °C successfully reduces the amount of aflatoxin produced. Some other common methods that are used to control aflatoxins in food include cooking, washing, steaming, broiling, and boiling. 

Treatment with ultraviolet radiation and ionization will result in cell wall degradation of the fungus, as well as a reduction in sprouting, which ultimately suppresses the growth of fungus; hence, controlling aflatoxin contamination in food and increasing the shelf life of food items [106]. Different thermal treatments can degrade aflatoxin concentration by 9% to 100%, depending upon the treatment used and the commodity to which the treatment is being applied, i.e., when fruits and other species are autoclaved at 120 °C for 30 min, this can degrade the aflatoxin concentration from 9–39%; whereas, if peanuts are autoclaved at 1.5 atm for 90 min, this can degrade aflatoxins by up to 100% [131]. 

Apart from thermal treatments, non-thermal treatment such as cold plasma may also be used to degrade aflatoxins by up to 95% in different grains and nuts [129]. Up to 97% aflatoxin decontamination has been reported by using irradiation, along with a detoxifying enzyme. The use of gamma rays effectively reduces aflatoxin contamination in fruits and vegetables by up to 60%. Aflatoxin contamination in milk can be reduced by treating animal feed with phospho-silicates, which primarily reduce aflatoxin contamination, causing a decrease in secondary aflatoxin contamination of milk. 

Various other techniques have been used, such as treatment with an adsorbent that detoxifies aflatoxins. The use of adsorbents can increase the shelf life of food, by reducing the production of secondary metabolites. Detoxification of aflatoxins such as B and G in food can be achieved by treating food items with sorbents, clays, and activated carbons. Different inorganic compounds, as well as their products such as hydrated sodium calcium aluminosilicates and phyllosilicates, bentonite, zeolite, and silicates, are also reported as successful detoxifying agents of aflatoxins. These compounds have ring-like structures and tetrahedrons, which consist of pores having electrical charges that trap aflatoxins [106].

## 7. Conclusions 

Aflatoxins are secondary metabolites produced by different species of *Aspergillus*, more specifically *A. flavus* and *A. parasiticus*, that are carcinogenic and toxic. The first studies on aflatoxins date back to the late 1950s and early 1960s. From that time till now, various studies and discoveries have been made regarding aflatoxins. There are 18 different types of aflatoxins that have long been discovered. New and advanced technology has also enabled mankind to study the structure of aflatoxins and their biosynthesis pathways utilizing different methods, which can be used to detect them at early stages. Different management strategies have also been employed for the control of these aflatoxins worldwide. This review will help researchers devise mitigating strategies, based on the information shared in this article.

## Figures and Tables

**Table 1:** Types of aflatoxin, producing fungal species, and commodities affected by aflatoxin (adapted from Ref. [11]).

Aflatoxin	Aflatoxin Producing *Aspergillus* spp.	Host/Affected Entity
B (B1, B2)	*A. arachidicola*, *A. bombycis*, *A. flavus*, *A. minisclerotigenes*, *A. nomius*, *A. ochraceoroseus*, *A. oryzae*, *A. parasiticus*, *A. parvisclerotigenus*, *A. pseudotamarii*, *A. rambellii*, *A. tamarii*, *A. toxicarius*, *A. versicolor*, *Emericella astellata*, *E. venezuelensis.*	Cottonseed, Dairy products, Figs, Fruit juices (apple, guava), Maize, Maize flour, Meat, Oilseed rape, Peanuts, Peanut butter, Pea, Pistachio, Rice, Sorghum, Sunflower seed, Spices
B2_a_	Hydroxylated metabolite of aflatoxin B1	-
B3 (Parasiticol)	Aflatoxin G1 metabolite, naturally produced by: *A. flavus*, *A. mottae*, *A. nomius*, *A. novoparasiticus*, *A. parasiticus*	Same as aflatoxin B1 and G1
G (G1, G2)	*A*. *arachidicola*, *A. bombycis*, *A. minisclerotigenes.* *A. nomius*, *A. parasiticus*, *A. pseudotamarii*, *A. terreus*, *A. toxicarius*, *A. versicolor*	Cottonseed, Dairy products, Figs, Fruit juices (apple, guava), Maize, Maize flour, Meat, Oilseed rape, Peanuts, Peanut butter, Pea, Pistachio, Rice, Sorghum, Sunflower seed, Spices
G2_a_	Hydroxylated metabolite of aflatoxin G1, also naturally produced by *A. flavus*	-
M (M1, M2)	Hydroxylated metabolite of aflatoxin B1 and B2, respectively	Dairy products, Milk, Meat
M2_a_	Aflatoxin M1 derivative	Dairy products, Milk
GM1	Hydroxylated metabolite of aflatoxin G1, naturally produced by *A. flavus*, also produced by *A. parasiticus* in vitro	Dairy products, Milk
GM2	Naturally produced by *A. flavus* and *A. parasiticus* and yeast, derived from aflatoxin G2	Dairy products, Milk
GM2_a_	Aflatoxin GM1 metabolite	Dairy products, Milk
P1	Metabolite ofaflatoxin B1 (demethylated)	Dairy products, Excreted in animals and human urine
Q1	Metabolite of aflatoxin B1 (hydroxylated)	Present in the meat of cattle that feeds on aflatoxin-contaminated food
Q2a	Acid hydration of aflatoxin Q1	-
Aflatoxicol R_0_	Metabolite of aflatoxin B1, also naturally produced by *A. flavus* and *A. parasiticus*	Present in bird feed, also exists in birds that feed on aflatoxin-contaminated food
Aflatoxicol M1	Aflatoxin B1, aflatoxin R_0_, or aflatoxin M1 metabolite	Dairy products, Milk
Aflatoxicol H1	Aflatoxin B1 and aflatoxin Q1 metabolite	Dairy products, Milk
Aspertoxin	*A. flavus* and *A. parasiticus*	Crops and Plants

**Table 2:** Primers sequence, their target genes, and the expected PCR product size.

S. No	Primer	Amplified Gene	Sequence	Size (bp)	References
1.	nor1	*nor*-1	5′-ACCGCTACGCCGGCACTCTCGGCAC-3′	400	[45]
	nor2	-	5′-GTTGGCCGCCAGCTTCGACACTCCG-3′	-	
2.	ver1	*ver*-1/*aflM*	5′-GCCGCACGCGGAGAAAGTGGT-3′	537	[45]
	ver2	-	5′-GGGGATATACTCCCGCGACACAGCC-3′	-	
3.	omt1	*omtA*/*aflP*	5′-GTGGACGGACCTAGTCCGACATCAC-3′	797	[46]
	omt2	-	5′-GTCGGCGCCACGCACTGGGTTGGGG-3′	-	
4.	Omt 208	*omtA*	5′-GGCCCGGTTCCTTGGCTCCTAAGC-3′	1024	[47]
	Omt-1232	-	5′-CGCCCCAGTGAGACCCTTCCTCG-3′	-	
5.	VER-496	*ver*-1	5′-ATGTCGGATAATCACCGTTTAGATGGC-3′	895	[47]
	VER-1391	-	5′-CGAAAAGCGCCACCATCCACCCCAATG-3′	-	
6.	APA-450	*apa*-2	5′-TATCTCCCCCCGGGCATCTCCCGG -3′	1032	[47]
	APA-1482	-	5′-CCGTCAGACAGCCACTGGACACGG-3′	-	
7.	aflR660	*aflR*	5′-CGCGCTCCCAGTCCCCTTCATT-3′	630	[45]
	aflR1249	-	5′-CTTGTTCCCCGAGATGACCA-3′	-	
8.	ord1501	*ord*1	5′-TTAAGGCAGGGGAATACAAG -3′	610	[48]
	ord2226	-	5′-GACGCCCAAAGCCGAACACAAA-3′	-	
9.	tub440-F	*ß-tubulin*	5′-GGTAACCAAATAGGTGCCGCT -3′	1300	[49]
	tub1740-R	-	5′-TAGGTCTGGTTCTTGCTCTGGATG-3′	-	
10.	nortaq-1	*nor*-1	5′-GTCCAAGCAACAGGCCAAGT -3′	66	[50]
	nortaq-2	-	5′-TCGTGCATGTTGGTGATGGT-3′	-	
	norprobe	-	5′-TGTCTTGATCGGCGCCCG-3′	-	
11.	aflR1-F	*aflR*	5′-AACCGCATCCACAATCTCAT-3′	798	[45]
	aflR1-R	-	5′-AGTGCAGTTCGCTCAGAACA-3′	-	
12.	Tub1-F	*tub*1	5′-GTCCGGTGCTGGTAACAACT -3′	1498	[45]
	Tub1-R	-	5′-GGAGGTGGAGTTTCCAATGA-3′	-	
13.	Nor1-F	*aflD*	5′-ACGGATCACTTAGCCAGCAC-3′	990	[51]
	NoR1-R	-	5′-CTACCAGGGGAGTTGAGATCC-3′	-	
14.	OmtB(F)-F	*aflO*	5′-GCCTTGACATGGAAACCATC-3′	1333	[51]
	OmtB(F)-R	-	5′-CCAAGATGGCCTGCTCTTTA-3′	-	
15.	Ord-gF	*aflQ*	5′-TTAAGGCAGCGGAATACAAG-3′	719	[51]
	Ord-gR	-	5′-GACGCCCAAAGCCGAACACAAA-3′	-	
16.	Omt1-F	*aflP*	5′-GCCTTGCAAACACACTTTCA-3′	1490	[52]
	Omt1R	-	5′-AGTTGTTGAACGCCCCAGT-3′	-	
17.	aflR-F1	*aflR*	5′-TGACCCACCTCTTCCCCCACG-3′	300	[51]
	aflR-R	-	5′-CCGTCAGACAGCCACTGGACACGG-3′	-	
18.	aflj-F/AP-F	*aflJ*	5′-AGTCAAAGGTTGAATACC-3′	840	[53]
	aflj-R/AP-R	-	5′-GCTCAGCCATGACCTTGACTG-3′	-	
19.	omtBII-F	*omt*-*B*	5′-ATGTGCTTGGGITGCTGTGG-3′	611	[54]
	omtBII-R	-	5′-GGATGTGGTYATGCGATTGAG-3′	-	
20.	AF138287	*ITS1-5*. *8rRNA*	5′-CTCCCACCCGTGTTTACTGT-3′	199	[55]
	AF027863	-	5′-GCGTTCTTCATCGATGCCT-3′	-	
21.	Asp1S	*5.8-28S rDNA*	5′-ATGCCTGTCCGAGCGT-3′	-	[56]
	AflR2	-	5′-TTAAGTTCAGCGGGTATRCCb-3′	-	
22.	AflP-F	*aflP*	5′-CATGCTCCATCATGGTGACT-3′	-	[4]
	AflP-R	-	5′-CCGCCGCTTTGATCTAGG-3′	-	
23.	FVAVIQ1	*ITS2 rDNA*	5′-GTCGTCCCCTCTCCGG-3′	-	[57]
	FLAQ2	-	5′-CTGGAAAAAGATTGATTTGCG-3′	-	
	PARQ2	-	5′-GAAAAAATGGTTGTTTTGCG-3′	-	
24.	cmd42^4^	*Calmodulin*	5′-GGCCTTCTCCCTATTCGTAA-3′	613	[58]
	cmd637^4^	*-*	5′-CTCGCGGATCATCTCATC-3′	-	
25.	cmd2F^3^	*Calmodulin*	5′-GGCTGGATGTGTGTAAATC-3′	811	[58]
	cmd2R^3^	*Calmodulin*	5′-ATTGGTCGCATTTGAAGGG-3′	-	
26.	niaDF^3^	*niaD*	5′-CGGACGATAAGCAACAACAC-3′	795	[58]
	niaDAR^3^	-	5′-GGATGAACACCCGTTAATCTGA-3′	-	
27.	niaDBF^3^	*niaD*	5′-ACGGCCGACAGAAGTGCTGA-3′	794	[58]
	niaDBR^3^	*niaD*	5′-TGGGCGAAGAGACTCCCCGT-3′	-	
28.	niaDCF	Nitrate reductase	5′-GCAGCCCAATGGTCACTACGGC-3′	-	[58]
	niaDCR	Nitrate reductase	5′-GGCTGCACGCCCAATGCTTC-3′	-	
29.	AP1729^5^	*norB-cypA*	5′-GTGCCCAGCATCTTGGTCCACC-3′	1839(no deletion) 903 (L strain) 323 (L+S strain)	[59]
	AP3551^5^	*-*	5′-AAGGACTTGATGATTCCTC-3′	-	
30.	CP-5F^6^	*norB-cypA*	5′-GGGACCCTTTTCCGGTGCGG-3′	3053(no deletion) 2134 (L strain) 1549(L+S strain) 836(LAF)^7^	[60]
	CP-R^6^	*-*	5′-GGCGGCCCCTCAGCAAACAT-3′	-	
31.	Taka-amylaseF^8^	*amyB/amy1*	5′-GGATCGATTTGCAAGGACGG-3′	1168	[61]
	Taka-amylaseR^8^	-	5′-TAGAGGTCGTCCATGCTGCC-3′	-	

**Table 3:** Aflatoxin biopesticides (registered and unregistered) along with the countries where they are being tested or used.

S. No	Product/Strain Name	Country	References
1.	AF36	U.S.	[97]
2.	Afla-Guard (strain NRRL21882)	U.S.	[98]
3.	CT3 (unregistered)	Southern U.S.	[99]
4.	K49 (unregistered)	Southern U.S.	[99]
5.	AF-X1	Italy	[100]
6.	Aflasafe SN01	Senegal and The Gambia	[101]
7.	Aflasafe GH01	Ghana	[102]
8.	Aflasafe GH02	Ghana	[102]
9.	Aflasafe	Nigeria	[103]
10.	Aflasafe KE01	Kenya	[103]
11.	AR27 (unregistered)	Northern Argentina	[104]
12.	AR100G (unregistered)	Northern Argentina	[104]
13.	AFCHG2 (unregistered)	Northern Argentina	[104]
14.	FS10 (unregistered)	China	[105]
15.	AF051 (unregistered)	China	[106]
16.	BN30 (unregistered)	Africa	[106]
17.	Aflasafe BF01	Burkina Faso	[107]
18.	Aflasafe TZ01	Tanzania	[108]
19.	Aflasafe TZ02	Tanzania	[108]
20.	Aflasafe ZM01 & ZM02	Zambia	[108]
21.	Aflasafe MW01 & MWMZ01	Malawi	[108]
22.	Aflasafe MZ01 & MWMZ01	Mozambique	[108]

**Table 4:** Aflatoxin gene clusters, markers for amplification, and their sequence and PCR product size. Adapted from Ref. [114].

S. no	Panel	Marker	Sequence	Size (bp)
01.	Sugar Cluster	SC01	5′-ATACCTCATGATCTGGTGCACGG 5′-CTTCGCAGCGACAATGATACGTC	883
02.		IC01	5′-GTCCCCAGGTACGATAGGTCTCT 5′-GCTGGATATTCCAAGGAGTGGCT	742
03.		AC01	5′-GACTGCCACCCTATCACTCTTCC 5′-TGGCTCGACTGGGTATGAAATCC	613
04.		AC02	5′-GCATTGCCAGCATCGGTTTCATA 5′-AGGCAGACCGTACTAAGTGATGC	487
05.		AC03	5′-CATGATGGAGCATGACATTCGGC 5′-GCGCCACCATATCTTCTCAGTCT	387
06.		AC04	5′-TTTAACCCTTCAYGCCTCGAACT 5′-TGCGTARCTAATCTCATCGGGTT	297
07.		AC05	5′-TGCTGAGCGAGTAGGTAGTAGGT 5′-CCGGATCATCCCTCCAAATCTGT	194
08.		iac	5′-GCTAGGGCGGGTCACGTTTTGCG 5′-GGCGTTGTTTAAGGGGAACCGACCC	115
09.	Aflatoxin Cluster	AC06	5′-CCTGTGAGGGACACAAAGACACT 5′-AAGAATAGCGGTGACATCCAGCA	1427
10.		AC07	5′-GAGGACAGGTTGTGTTGCTGTTG 5′-GTTCACGAGCTATCCTCAGCCAT	1092
11.		AC08	5′-GAACTGAGCCATTTCCATCAGCG 5′-GTCTTGTACAGGGAACGTGGTGA	897
12.		AC09	5′-AACGCTTCAACGTGGAGGACATA 5′-AATAGCGTTGGCGTTGAAGTCAC	736
13.		AC10	5′-CCCGCATTTTTCTCGATCCCTTG 5′-GCGACGACCAGTCATTATGAAGC	633
14.		AC11	5′-GTCAGACCACAGTGAGTGCTTCT 5′-AAGCTGACTGGGAGAATGTTGCT	536
15.		AC12	5′-CCCCTCAACTTCTGTCGTCCTAC 5′-GCTGGGTAGCGAACAATCCAATG	425
16.		AC13	5′-GCACACAGCAGAGGCATTTCTAC 5′-AATCTATCTAGCCATCGCCACCG	330
17.		IC02	5′-GCCTGCTAGGCTTGGAACTATGT 5′-CGCAATGCTAGTATGCCCTTGTC	209
18.		iac	5′-GCTAGGGCGGGTCACGTTTTGCG 5′-GGCGTTGTTTAAGGGGAACCGACCC	115
19.	CPA Cluster	CC01	5′-GACACTCGTACCATCTATGCACC 5′-GATCCCTGATCCATTCCACCTTG	1219
20.		CC02	5′-ACGATACGAGCTTTAGTGCAAGG 5′-GATATAGACCTCAGGGTCGAGCA	925
21.		CC03	5′-AGAGCTGCGCACTCCATTT 5′-TGCCCAGGCAATAGGAAGTA	821
22.		CC04	5′-ACCTCAACAATTACACCGGATGG 5′-GTTGTAGCTCAACGTCACTAGCA	648
23.		ST01	5′-TATCTATCTGGGATACGGGCTGG 5′-TATGCCGTTGCTATCCAATGAGG	521
24.		ST02	5′-AAGTCAGATTCCGCGGTATGAAG 5′-TCATCGCATTAATCGAGGCAGTT	416
25.		ST03	5′-CCTCCTGCACAAAAATACTCCCA 5′-GATCAGATCTTTGAGCGTAGCGT	320
26.		ST04	5′-TCATGTTTCGGATCGGAGATTGG 5′-ACATTCCAAGTGAGAGATGTGGC	234
27.		iac	5′-GCTAGGGCGGGTCACGTTTTGCG 5′-GGCGTTGTTTAAGGGGAACCGACCC	115
28.	Sub-telomere	ST05	5′-ACTGGTGTTGGATAGAGCTCAGA 5′-TGGAAGGTTCTCCGGATACTTGA	908
29.		ST06	5′-TACTCCGTTGCTGTCATTGGATG 5′-CGAATTCTTGGTTGAGCAGCTTG	782
30.		ST07	5′-TGCTGAATAACAACCTCGACCAG 5′-CAGGCTGGTATAGCACCAATGTT	684
31.		ST08	5′-GGTTTCGTCTTGCCTTCTTCTCA 5′-AGCAAAGTGATGCCGTTCAAATG	584
32.		ST09	5′-CGTACTTTGTTACGGCGTACATC 5′-GCTGTTTCGCGTTAGTTGGTAAC	512
33.		ST10	5′-GCCCGTAAATGAGGTGCAGATAA 5′-TTTGGGTGTGCTTCTTCATGCTA	404
34.		ST11	5′-GGGGACTTAGTCGCGAATGGTTA 5′-TATGAAGGCCACCAACTGAGGAC	285
35.		ST12	5′-AATGACGACACTTGAGGCACAG 5′-TCGGCTCCGTGACACCATATTA	185
36.		iac	5′-GCTAGGGCGGGTCACGTTTTGCG 5′-GGCGTTGTTTAAGGGGAACCGACCC	115

## Data Availability

The data presented in this study are available in this article.
